# Graph rigidity reveals well-constrained regions of chromosome conformation embeddings

**DOI:** 10.1186/1471-2105-13-241

**Published:** 2012-09-21

**Authors:** Geet Duggal, Carl Kingsford

**Affiliations:** 1 Department of Computer Science and Institute for Advanced Computer Studies University of Maryland, College Park, MD 20742, USA

## Abstract

**Background:**

Chromosome conformation capture experiments result in pairwise proximity measurements between chromosome locations in a genome, and they have been used to construct three-dimensional models of genomic regions, chromosomes, and entire genomes. These models can be used to understand long-range gene regulation, chromosome rearrangements, and the relationships between sequence and spatial location. However, it is unclear whether these pairwise distance constraints provide sufficient information to embed chromatin in three dimensions. A priori, it is possible that an infinite number of embeddings are consistent with the measurements due to a lack of constraints between some regions. It is therefore necessary to separate regions of the chromatin structure that are sufficiently constrained from regions with measurements that do not provide enough information to reconstruct the embedding.

**Results:**

We present a new method based on graph rigidity to assess the suitability of experiments for constructing plausible three-dimensional models of chromatin structure. Underlying this analysis is a new, efficient, and accurate algorithm for finding sufficiently constrained (rigid) collections of constraints in three dimensions, a problem for which there is no known efficient algorithm. Applying the method to four recent chromosome conformation experiments, we find that, for even stringently filtered constraints, a large rigid component spans most of the measured region. Filtering highlights higher-confidence regions, and we find that the organization of these regions depends crucially on short-range interactions.

**Conclusions:**

Without performing an embedding or creating a frequency-to-distance mapping, our proposed approach establishes which substructures are supported by a sufficient framework of interactions. It also establishes that interactions from recent highly filtered genome-wide chromosome conformation experiments provide an adequate set of constraints for embedding. Pre-processing experimentally observed interactions with this method before relating chromatin structure to biological phenomena will ensure that hypothesized correlations are not driven by the arbitrary choice of a particular unconstrained embedding. The software for identifying rigid components is GPL-Licensed and available for download at http://cbcb.umd.edu/kingsford-group/starfish.

## Background

Recent experiments for chromosome conformation capture [1-7] can result in graphs of hundreds of thousands interactions between chromosome locations. Each edge in such a *chromosome conformation graph* is associated with a weight corresponding to the frequency at which the interaction occurs, and the edges in the graph can be interpreted as spatial distance constraints between chromosome locations with an appropriate mapping from interaction frequency to distance [2-4]. The information contained in chromosome conformation graphs has been used to embed entire genomes as well as portions of chromosomes at a kilobase-pair resolution in three dimensions [2-5,7,8], and these structures provide first glimpses into how chromosomes take shape within the cell in more detail than what is possible with light microscopy [9]. These experiments are also motivated by the potential to associate genome structure with long-range regulation, chromatin accessibility, and somatic copy number alterations [10]. Embedding chromosome conformation data has become a common practice, and a variety of algorithms have been developed to embed these structures in three dimensions [2,4,11]. These embedded structures have been used to gain biological insight into how chromatin structure relates to cancer [4], how sequence relates to to structure [7], and to study chromatin territories [5].

Our primary objective is to determine whether chromosome conformation data from recent experiments on the budding yeast, fission yeast, and human genomes provide an adequate set of constraints for embedding confidently. Underconstrained, *floppy* substructures of an embedded genome can continuously deform without violating any measured distance constraints, resulting in an infinite number of embeddings consistent with the experimental data. As a pre-processing step before embedding, it is thus desirable to identify non-floppy or *rigid* substructures within the genome. It is these structures for which we have the most confidence in three-dimensional embeddings provided by optimization methods such as described in [2-4]. Rigid regions are not rigid in the sense of being physically frozen. In fact, a rigid region can be asssociated with a variety of unique embeddings consistent with distance constraints in the conformation graph. In addition, chromosome conformation measurements at various time points may reveal other snapshots of chromatin structure, and this ensemble of embeddings can reflect the highly flexible nature of chromatin. In contrast, if a substructure of chromatin is not rigid, the flexibility is simply due to the fact that the region is underconstrained by the experimental measurements. Filtering subsequent spatial analyses to consider only those regions that are rigid will help to avoid artifacts created merely by the lack of sufficient constraints to select among consistent, continuously deformable alternatives.

We apply graph rigidity theory [12,13] to determine the substructures within the genome that are sufficiently constrained to produce a non-floppy embedding in three dimensions. Two key features of our technique are that it deals directly with the chromosome conformation graph rather than relying on computing a spatial embedding and that it does not depend on the precise values of the distance constraints. These are both highly desirable properties for assessing the quality of chromosome conformation data for embedding because there is no consensus yet on a mapping from frequency to distance and computing even a single spatial embedding can be computationally very expensive for an entire genome. In order to efficiently assess rigidity on the scale required by the chromosome conformation capture data, we propose a novel, fast algorithm for identifying rigid substructures. This algorithm uses a family of “pebble game” algorithms [14-17] established for finding rigid substructures in tandem with a novel algorithm using results from rigidity theory [18]. Under the assumption that the edges in these graphs represent fixed distance constraints, the proposed algorithm guarantees that all subgraphs identified are rigid in three dimensions, although they may not be maximal.

While it could be the case that significant portions of the constraints are floppy and potentially uninformative for embedding, we find that, for even strictly filtered graphs, a large rigid subgraph that spans most — but not all — of the genome. Thus, since the region is not underconstrained, the embedded structures of most regions can be more confidently interpreted. This procedure can be applied to any statistical filtering of chromosome conformation data, and we explore the effect of filtering both low-frequency and short-range interactions on the creation of rigidly embeddable structures. Most interactions in genome-wide chromosome conformation graphs occur either infrequently or at short genomic distances, and some of these interactions could be a result of experimental noise or arise from incidental, transient interactions. By systematically filtering interactions, we quantify the frequency cutoff at which large rigid components begin to disappear. Additionally, we find that the creation of rigid components depends crucially on short-range intra-chromosomal interactions and that the pairing or separation between rigid, subtelomeric regions of chromosomes is consistent with light microscopy data for budding and fission yeast.

## Results and discussion

### Algorithms for identifying rigid components

Rigid components correspond intuitively to substructures in the embedding that cannot be continuously deformed without violating one or more measured proximities between chromosome locations. Formally, a graph of distance constraints is a *rigid graph* or *rigid body* in three dimensions if, when the vertices are embedded in generic position in R^3^, there is no continuous movement of the vertices — aside from a rotation or translation of all vertices — that maintains all the distances between vertices connected by edges. If a graph is not rigid (i.e. *floppy*), infinitely many embeddings are possible since there exists at least one continuous movement of vertices that maintains all the distance constraints. A *rigid component*, or maximally rigid subgraph, is a subset of vertices *C* for which the subgraph induced by *C* is rigid and no superset D⊃C exists for which the subgraph induced by *D* is rigid. We only consider rigid components with 3 or more nodes, although vertices with no edges and single edges can be trivial rigid components of size 1 and 2 respectively.

**Figure 1:**
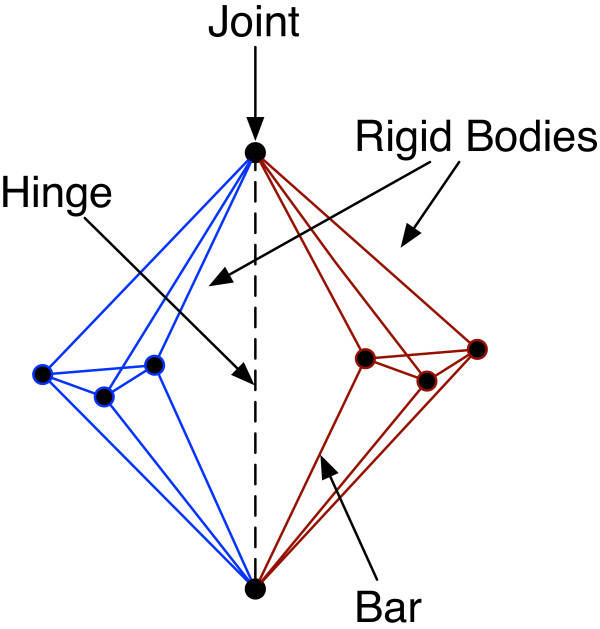
**The double-banana graph.** The dotted line represents an implied axis of rotation.

There are several related notions of rigidity, depending on the types of motions allowed. In a general *bar-joint framework*, vertices represent universal *joints* and edges represent fixed-length *bars* between joints. The double-banana graph (Figure 1) is composed of two rigid components in this framework that rotate around a hinge implied by two joints in the graph. The double-banana can also be represented as a type of bar-joint framework called a *body-bar-and-hinge framework* where rigid bodies can be connected to one another by fixed-length bars as well as hinges that allow just one rotational degree of freedom between two rigid bodies. The double-banana is also an example of a graph that contains rigid components that share nodes, illustrating the fact that rigid components of a graph do not correspond necessarily to a partition of the vertices in the graph.

No efficient algorithm is known for identifying all rigid components in three dimensions in general bar-joint frameworks. Efficient algorithms based on the so-called “pebble games” do exist in two dimensions [14,15] and for more restricted notions of rigidity in 3-dimensions [13]. Recently, it has been suggested that a variant of a pebble game algorithm designed for two-dimensional rigidity can be applied to arbitrary bar-joint frameworks in three dimensions [13] with good results for most graphs. While this approach often identifies many rigid components, it also erroneously produces components that are floppy. One such example is the double-banana graph of Figure 1. In contrast, efficient, provably correct algorithms exist to find rigid components in body-bar-and-hinge frameworks [16].

We propose an iterative procedure we call Body-Bar-and-Hinge Reduction (Algorithm 1) for more accurately finding rigid components in three dimensions. It begins by gluing together smaller rigid subgraphs and then merges them by reducing the problem to identifying rigid components in the body-bar-and-hinge framework, for which efficient algorithms exist. For graphs close to the minimally rigid threshold (3*n*−6 edges where *n* is the number of vertices in the graph), we suggest the use of a hybrid algorithm (Algorithm 2) that combines the pebble game with the body-bar-and-hinge reduction. In this variant, whenever the pebble game returns a floppy component, Algorithm 1 is run on the component. The pebble game fails when implied hinges exist such as the one in the double-banana graph [13]. In these cases, we observe the pebble game over-estimates the size of the actual rigid components and Algorithm 1 decomposes this floppy component into rigid subgraphs.

#### Algorithm 1

**Body-Bar-and-Hinge Reduction** Let Max-Triangle(*G,U*) and Max-Vertex(*G,U*) be a triangle or vertex in *G*, respectively, with the largest total degree excluding edges incident to vertices in *U*. 

1: **Input:** A graph *G* of distance constraints

2: Remove all vertices of degree ≤ 2

3: Initialize the list of rigid subgraphs R to the empty list

4: **while** a $T=\text{Max-Triangle}(G,\underset{C\in R}{⋃}C)$ can be found such that *T* is not fully contained in any component in R**do**

5:  **while** a $v=\text{Max-Vertex}(G,\underset{C\in R}{⋃}C)$ with *v*∉*T*and at least three edges to *T* can be found **do**

6:   Add *v* to *T*

7:  Add *T* to R

8: **while** two components $C_{i},C_{j}\in R$ share three or more vertices **do**

9:  Remove both *C*_*i*_and *C*_*j*_from R

10:  Add $C_{i}\cup C_{j}$ to R

11: Let $R_{2}$ be a subset of R such that for each pair *C*_*i*_,*C*_*j*_in R, $|C_{i}\cap C_{j}|=$ 0 or 2.**Comment:** The body-bar-and-hinge framework will be represented by a set of hinges *H* which contains pairs of rigid bodies that share two vertices and a set of bars *B* which contains edges that connect rigid bodies.

12: Initialize *B*, *H*, a set of used hinges *U*_*H*_, and a set of used nodes *U*_*N*_to the empty set.

13: **for** every pair *C*_*i*_,*C*_*j*_in $R_{2}$**do**

14: **if**$|C_{i}\cap C_{j}|=2$ and $C_{i}\cap C_{j}=\{v,w\}\notin U_{H}$**then**

15:  Add {*C*_*i*_,*C*_*j*_} to *H*

16:  Add {*v*,*w*} to *U*_*H*_

17:  Add both *v* and *w* to *U*_*N*_

18: **for** all pairs of nodes *v*,*w*in *C*_*i*_△*C*_*j*_**do**

19:  **if***G* contains an edge between *v* and *w*, *v*∉*U*_*N*_, and *w*∉*U*_*N*_**then**

20:   Add {*v*,*w*} to *B*

21:   Add both *v* and *w* to *U*_*N*_

22: **Return:** the subsets of vertices in *G* corresponding to the rigid components of the body-bar-and-hinge framework as well as components in $R\setminus R_{2}$

#### Algorithm 2

Identify Rigid Components

1: **Input:** A graph *G* of distance constraints

2: Initialize the list of rigid components C to the empty list

3: **for** every connected component *G*_*i*_in *G***do**

4: Let P be the set of components for *G*_*i*_returned by the pebble game algorithm

5: **for**H∈P**do**

6:  **if** the subgraph induced by *H* is floppy **then**

7:   append all components returned by Body-Bar-and-Hinge Reduction on the subgraph induced by *H* to C

8:  **else**

9:   append *H* to C

10: **Return:**C

To determine whether a component produced by the pebble game is floppy or rigid (line 6 of Algorithm 2), we use the standard rank test of a matrix that encodes a graph of distance constraints given an embedding in $R^{3}$[12]. If a random embedding of a graph of distance constraints is rigid, then all generic embeddings are also rigid [19]. This fact allows the rigidity of an identified subgraph of distance constraints to be tested via random embeddings, ignoring the precise distances on the constraints.

We construct rigid subgraphs using Algorithm 1, which starts greedily from a triangle with the most connections to other vertices not yet in a rigid component. This rigid subgraph is then grown one vertex at a time such that each added vertex connects to at least three vertices in the existing subgraph and has the most connections to other vertices not in the subgraph (lines 3-6). By Proposition 1, the grown subgraph is rigid. Once no vertex can be added, another triangle not contained in an existing component is selected and grown by the same vertex addition allowing reuse of any vertex added in a prior step. Once no more triangles can be found, constructed rigid subgraphs that overlap by three or more vertices are merged to form larger rigid subgraphs (lines 8-10). Proposition 2 below guarantees that components merged in this way will be rigid.

#### Proposition 1

If a vertex connects to at least three nodes in a rigid subgraph, then extending the subgraph to include that vertex results in a rigid subgraph. (Vertex 3-Addition Lemma[18])

#### Proposition 2

If two rigid subgraphs overlap by 3 or more nodes, then the union of the subgraphs is rigid (Generic 3-Gluing Lemma[18]).

The resulting subgraphs are merged further by converting them into a body-bar-and-hinge framework as described in lines 11-21 of Algorithm 1.

#### Proposition 3

Algorithm 1 returns rigid components.

By Propositions 1 and 2, the subgraphs produced by the initial greedy phase of Algorithm 1 are rigid and can be used as bodies. Line 11 eliminates the possibility that pairs of rigid bodies overlap by exactly one node: this overlap can neither be represented as a hinge between two rigid bodies nor a bar between two distinct vertices. The framework is then constructed by assuring that each hinge connects exactly two rigid bodies that overlap by two vertices. Lines 14-17 guarantee that whenever a hinge is created between a pair of rigid bodies that overlap by two vertices, that pair of vertices is never used as a hinge again. Lines 18-21 similarly assure that vertices across two rigid bodies are connected together by bars such that no vertex contains multiple bars. These basic rules construct a body-bar-and-hinge framework where hinges only allow one degree of rotational freedom between two rigid bodies and that bars do not share end points [20]. Rigid components in this framework directly correspond to rigid components in the original graph. By a theorem of Tay [21], a variant of the pebble game can be used to identify rigid components in body-bar-and-hinge frameworks, and this can be done in time quadratic in the number of vertices [16].

**Figure 2:**
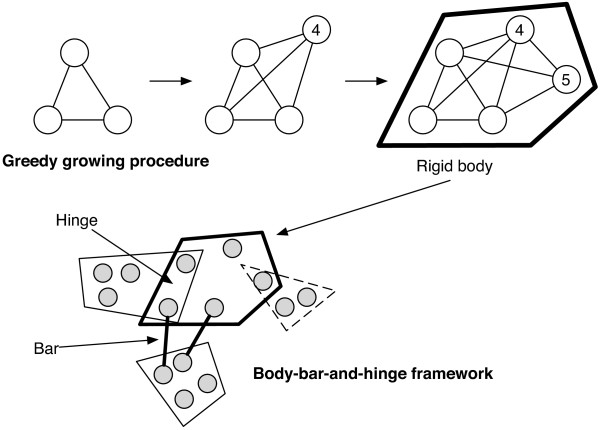
**Schematic of the body-bar-and-hinge reduction.** The rigid body in the dotted line is not included since it does not form a hinge with another body and no bar connects it to another body.

Although, by Proposition 3, the subgraphs produced by Algorithm 1 are rigid, they may not be maximally rigid subgraphs (i.e. rigid supergraphs may exist). This is because certain bar-joint frameworks cannot be represented as body-bar-and-hinge frameworks (e.g. two triangles with a shared node), and therefore some rigid components may be missed. However, the algorithm proposed here correctly identifies the two rigid components in the double-banana, which are incorrectly merged by the pebble game in three dimensions. Figure 2 illustrates the technique.

For graphs close to the minimally rigid threshold (3*n*−6 edges in three dimensions where *n* is the number of vertices in the graph), Algorithm 1 may fail to identify the maximal rigid component. In these cases, we propose using a hybrid algorithm (Algorithm 2) that combines the body-bar-and-hinge reduction with the pebble game algorithm. Since the pebble game does not guarantee that the components it returns are rigid, Algorithm 2 performs matrix rank tests on these components to verify that they are indeed rigid. The bottleneck of Algorithm 2 is the matrix rank testing of components returned by the pebble game, which takes *O*(*mn*^2^) time, where *m* is the number of edges in the graph and *n* is the number of vertices.

### Performance of rigid component algorithms

Although there is no known algorithm that efficiently identifies all maximally rigid subgraphs of bar-joint frameworks in three dimensions at this scale, for a few small individual chromosomes in budding yeast (1,2 and 6) at interaction frequency cutoffs of 98.8, 99.0, 99.2, and 99.4% (see Methods), we observe that Algorithm 1 finds maximally rigid subgraphs. To verify that we find a maximally rigid subgraph, we performed matrix rank tests on all possible induced subgraphs with more vertices than the largest rigid component identified by Algorithm 1. We also compared Algorithm 1 with a recently proposed slow spring relaxation algorithm [13] and found identical rigid components.

For even a single chromosome, the exhaustive subset testing technique takes hours to days on 20 Opteron 8431 (2400MHz) processors and the spring relaxation algorithm takes a similar amount of time on a single processor. A rigidity analysis using these techniques would be infeasible, but Algorithm 1 can identify rigid components on the entire yeast genome (Duan et al. with their FDR 0.01% filtering) in minutes on a single processor. This is despite the fact that finding the maximum triangle, which takes *O*(*n*^3^) time, is the bottleneck in Algorithm 1. On the other hand, finding any triangle in a graph is at most the time complexity of a matrix multiplication [22]. If we replace the greedy requirement of finding a maximum triangle and maximum vertex with finding any triangle or vertex that meets the edge connection criteria, we obtain identical results at much lower running times (<20 seconds for the Duan et al. genome at FDR 0.01%). In addition, when comparing Algorithm 1 to the pebble game for bar-joint networks, we find identical rigid components for all individual chromosomes in the Duan et al. data set. The pebble game algorithm alone runs in similar time to Algorithm 1, but doesn’t guarantee rigidity. When rank tests are used to confirm rigidity for the pebble game algorithm, the running times are at least 20 times the running times without the rank tests.

### Rigid components in augmented vs. non-augmented chromosome conformation graphs

**Figure 3:**
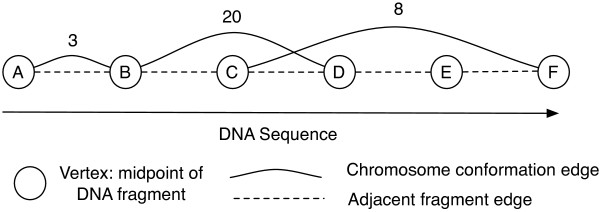
**Example chromosome conformation graph.** Example augmented chromosome conformation graph. Each node represents a chromosome location and edges represent distance constraints.

Augmented chromosome conformation graphs explicitly incopropate constraints to model the linear nature of chromatin (see Methods). Adding constraints between betweeen adjacent fragments can increase the sizes of the rigid components in the graph. For example, in Figure 3 the addition of these edges causes vertices B, C, and D to form a triangle, which is rigid. Vertices not observed in the experiment have degree ≤2 since the edges between adjacent components form a path in the graph. Since any vertex of degree ≤2 cannot contribute to a rigid component, vertices not observed in experiment do not change the rigid components in the graph (e.g. vertex E in Figure 3).

The pebble game obtains larger rigid components than Algorithm 1 when maximally rigid subgraphs are close to the minimum number of edges required for rigidity (3*n*−6 edges in three dimensions where *n* is the number of vertices in the graph), and Algorithm 2 will always find rigid components at least as large as the pebble game since floppy components returned by the pebble game are decomposed into smaller rigid components, and by Proposition 3, it will never report a floppy component as rigid. Algorithm 2 uses the pebble game in two ways: first, a version of it [13] is applied directly to other input network and, after rigidity matrix tests, if any of these components are floppy, Algorithm 1 is applied using a version of the pebble game to find components on the body-bar-and-hinge network. This version of the pebble game explicitly models the bars and hinges in the body-bar-and-hinge framework. Further discussion and demonstrations of the pebble game and its application can be found online [17].

We find that augmented constraints may be useful when embedding the data, but they are not required for obtaining large rigid substructures. Even though the augmented chromosome conformation graph can add many new edges (e.g. around 4,000 for Duan et al.), for all genomes, the size of the largest rigid component increases by no more 5% (Table 1). This suggests that there are enough short-range interactions in the experimental data so that constraints between adjacent fragments are redundant when determining whether the graph is rigid.

**Table 1:** Largest rigid component sizes for genome-wide experiments

	**Unaugmented**		**Augmented**	
**Experiment**	**Graph size**	**Rigid component**	**Graph size**	**Rigid component**
GM06690	2,880	2,879	2,882	2,880
K562	2,874	2,874	2,882	2,874
Budding yeast	3,172	2,880	4,193	2,959
Fission yeast	611	590	619	606

The number of vertices (graph size) in the largest connected component and the number of vertices in the largest rigid component for genome-wide chromosome conformation graphs (unaugmented and augmented) at the 98.8% interaction frequency cutoff.

### Effect of low-frequency and short-range interactions on rigid components

**Figure 4:**
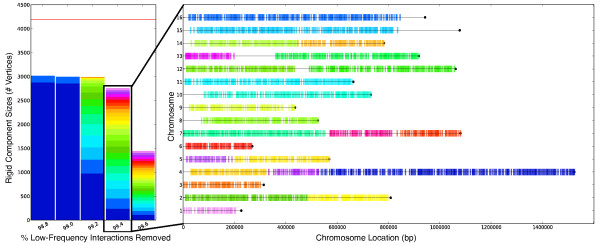
**Effect of low-frequency interactions on rigid components.** (Left) Sizes of rigid subgraphs after removing various percentages of low-frequency interactions for the Duan et al. chromosome conformation graph. The rigid subgraphs at a particular cutoff are sorted and colored by size. The horizontal red line represents the total number of nodes in the chromosome conformation graph before filtering. (Right) The chromosomal locations of rigid components after removing 99.4% of low-frequency interactions. Bars indicate centers of the fragments involved in a rigid component, and colors indicate the various components.

Running Algorithm 2 on filtered chromosome conformation data for the fission yeast, budding yeast, and human genomes results in one large rigid component for each genome (Table 1). We apply stringent filters since most interactions occur very infrequently and we wish to determine the rigidity of the experiments from the most probable highest-confidence interactions. Although rigid graphs can be very sparse (i.e. rigid components are not necessarily dense graphs), denser graphs are more likely to be rigid. However, even after removing more than 98% of the low-frequency interactions, a single large rigid subgraph comprising most of the genome is found. For Duan et al. (Figure 4, left), even after removing 98.8% of the low-frequency edges, a rigid component with nearly three-fourths of all possible nodes is obtained (the horizontal red line in Figure 4 represents the total number of nodes in the conformation graph). The density of this subgraph is nearly one-third the density of the most stringently filtered set of interactions provided by Duan et al. and each edge in the subgraph has an observed interaction frequency ≥ 30. Rigidity analysis directly on their filtered data also produces a single, large rigid component. Notably, the fission yeast conformation graph of Tanizawa et al. is rigid despite being close to the minimum number of edges required for rigidity: at a cutoff of 98.8%, there are 611 nodes and 2,167 edges, just 340 more edges than are necesssary for the graph to be rigid. This shows that, even after stringent filtering of interactions, there is sufficient data to restrict most of the genome to only a finite set of possible embeddings.

**Figure 5:**
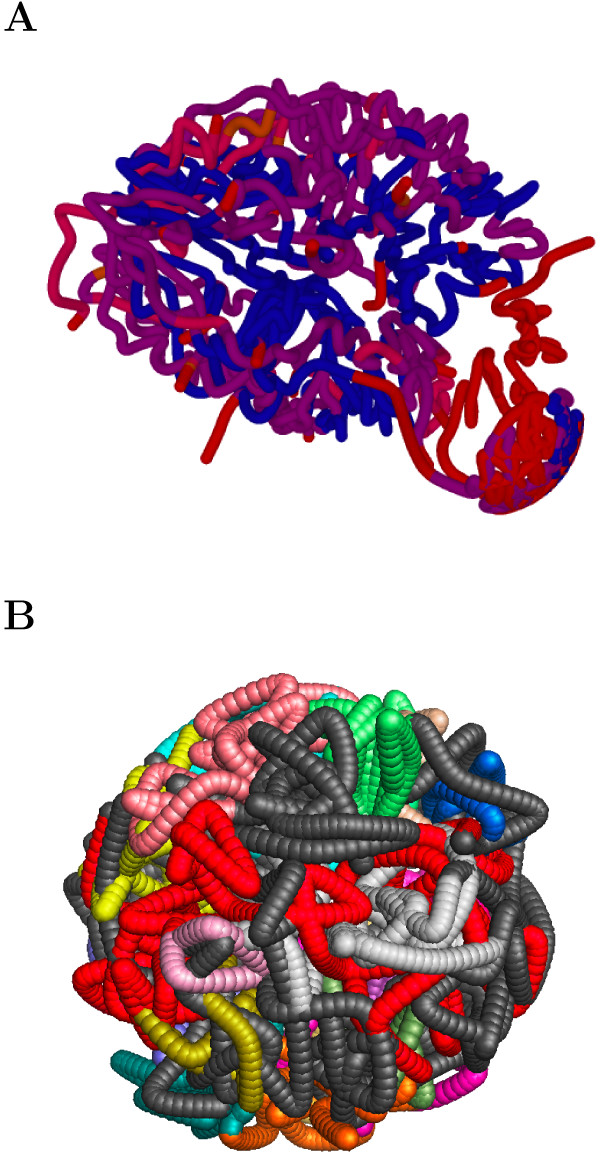
**Highlighting structures with rigidity analysis. (A)** Confidence in the embedding of the Duan et al. structure. Segments of the genome are colored according to the interaction frequency cutoff at which the segment becomes floppy. Red regions correspond to the 98.8 cutoff% and blue regions are still rigid at the 99.6% cutoff. **(B)** The Tanizawa et al. structure colored by rigid component for interaction frequency cutoff 99.0%. Dark gray indicates floppy regions. Rigid components in the subtelomeric regions of chromosome 1 are red (see Discussion).

As more low-frequency interactions are removed, the original component breaks apart into multiple rigid components that still span most of the genome (Figure 4, right). The rigid components are usually subgraphs of connected components of the filtered graph, not entire connected components themselves. Figure 5(A) shows the Duan et al. embedding colored by the interaction frequency cutoff at which a segment of the genome becomes floppy. Even after removing 99.6% of the low-frequency interactions, nearly one-third of the embedding remains rigid. Figure 5(B) highlights rigid components at the 99.0% interaction-frequency cutoff for the fission yeast genome.

**Figure 6:**
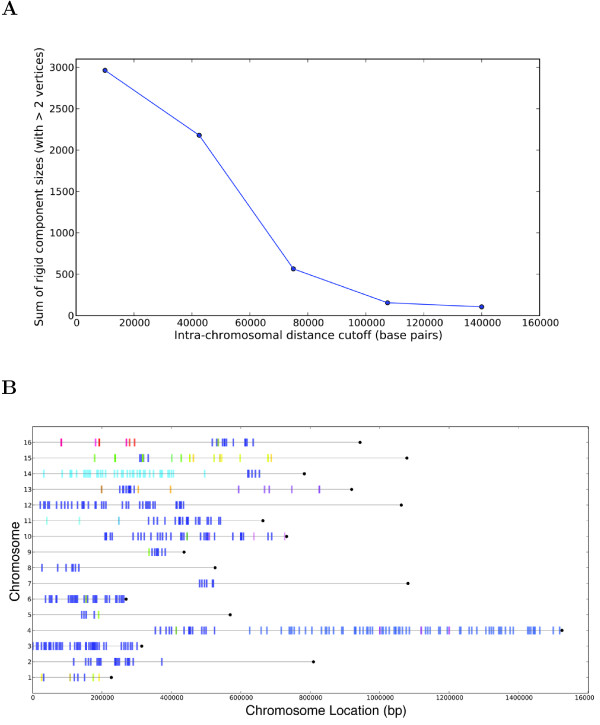
**Rigid components after removing short-range interactions. (A)** Sum of rigid component sizes after removing all interactions below increasing intra-chromosomal distances (98.8% frequency cutoff). **(B)** Chromosomal locations of rigid components after removing intra-chromsomal interactions that occur within 75kbp for the Duan et al. chromosome conformation graph (98.8% frequency cutoff). Bars indicate centers of the fragments involved in a rigid component, and colors indicate the various components.

By systematically removing short-range, intra-chromosomal interactions on frequency-filtered graphs (i.e. all those below some chromosomal distance), we find that such interactions (i.e. typically those below 40 kbp) are crucial for maintaining a large rigid component comprising most of the genome. Figure 6, for example, shows that removing interactions that span ≤75 kbp results in the elimination of nearly all large rigid components. It shows that the rigid embeddability of the chromosome conformation data depends centrally on these short-range contacts to provide a backbone of constraints for genome-wide chromosome conformation data sets. This dependency on short-range interactions holds for all data sets except Bau et al. which is targeted to a small region of human chromosome 16 and still maintains a large rigid component (with at least half of all possible vertices) even after removing all interactions ≤140 kbp. The fact that both the non-cancer and cancer data sets of Bau et al. preserve large rigid components after stringent filtering of short-range interactions may be due to the fact that the Bau et al. data set is of higher quality and has larger interaction frequencies for both cell types.

### Rigid components of a graph filtered for metric distances

An alternative way to filter the experimental data is to keep only those constraints that satisfy the metric properties under some frequency to distance mapping. Since chromosome conformation graphs are an aggregation of interactions from millions of cells, each with some conformation of chromatin, it is possible that dense subgraphs resulting from this aggregation are associated with proximities that contradict one another when attempting an embedding. For example, any clique with >4 nodes where the distance between any two nodes is required to be the same is impossible to embed in three dimensions. In general, the problem of determining whether a graph of distance constraints can be embedded in three dimensions is NP-hard [23]. However, one necessary condition for a graph to be embedded in three dimensions is that all interactions satisfy the metric properties.

We therefore also tested a filtering scheme that keeps only sets of edges that satisfy the triangle inequality. This is yet another stringent filtering applied to the data set to test for rigidity. Consider a chromosome conformation graph where weights on the edges are defined to be the distance as determined by a frequency to distance mapping [2-4]. We obtain the set of interactions {*uv*} in the subgraph with lengths equal to the weighted shortest path between *u* and *v*; this set satisfies the shortest path metric. The Duan et al. subgraph (FDR 0.01%) after this metric filtering still contains 3,525 vertices and 27,301 edges and one large rigid component with 2,987 vertices. Therefore, even after including only high-frequency, metric interactions, there is sufficient data to obtain a nondeformable embedding.

### Discussion

**Figure 7:**
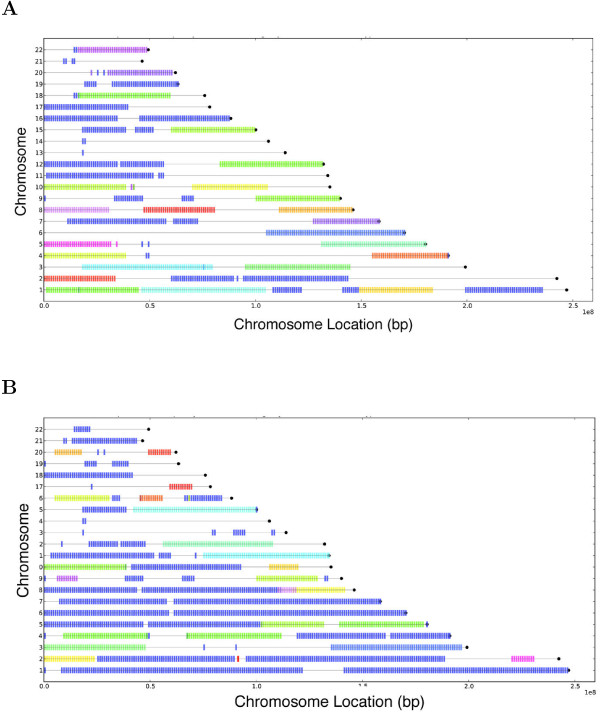
**Rigidity of cancer vs. non-cancer graphs.** Chromosome locations of rigid components for Lieberman-Aiden et al. **(A)** lymphoblastoid cell and **(B)** cancer cell colored by rigid component for interaction frequency cuttoffs 99.4%.

Although our primary objective is to determine the extent to which currently available chromosome conformation data is rigid, we additionally choose specific cutoffs (since there is no established cutoff) and anecdotally discuss the properties of rigid components in these contexts. For example, the set of rigid components for the two human cell types differ significantly: at a 99.4% cutoff, the largest rigid component of the cancer cell covers all of chromosomes 1, 6, and 7, while the largest rigid component of the healthy cell covers only chromsome 16 despite the fact that they each have a similar number of vertices and edges (Figures 7(A) and 7(B)). Cancer and non-cancer cell types are commonly compared in 3C experiments (e.g. [1,4,10]), and assuming this more stringent filter is used for an embedding, this analysis suggests it is possible to confidently embed a much larger portion of the cancer genome than the normal lymphoblastoid genome. While it is desireable to compare the entire structures, structural comparisons can more confidently be made between mutually rigid subsets of the genome at this cutoff, and focusing comparisons on mutually rigid subsets guarantees that the difference between structures is not due to the fact that one or both of them is underconstrained. The large difference in rigid component sets may be due to the fact that the cancer and non-cancer genomes are structurally very different from one another. The Bau et al. study establishes this for a small portion of chromosome 16 with both chromosome conformation data and microscopy experiments, but there has been no genome-wide structural comparsion of these cell types.

Microscopy data also confirms some observed properties for the rigid components in the Duan et al. and Tanizawa et al. data sets. At the 99.4% and 99.6% interaction frequency cutoffs, the larger chromosomes in budding yeast break apart into multiple large rigid components (Figure 4, right) with subtelomeric regions in different rigid components. This is consistent with the fact that the subtelomeric regions of chromosomes 4, 12, and 13 are known to be separated from one another and near the nucleolus and nuclear periphery [24,25]. For chromosome 12 of budding yeast, a subtelomeric region containing ribosomal DNA close to the nucleolous is a part of its own rigid component even at a 98.8% interaction frequency cutoff [2]. For chromosome 1 of the fission yeast genome (interaction frequency cutoff 99.0%), the subtelomeric regions at each end are part of a single rigid component (the red region in Figure 5(B)) and these regions are also observed in close proximity to one another in microscopy experiments [3,26].

To capture the space of possible structures, our rigid components algorithm can also be used as input to a recent technique that creates an ensemble of embeddings from chromosome conformation data [11]. Generating an ensemble of embeddings can be slow on large collections such as [1], and a potential speedup can be achieved by randomly permuting the edges of the input graph passed to the pebble game. This procedure samples the minimimally rigid subgraphs built with the pebble game. Although it is unlikely that any embedding represents a structure that existed for any particular cell, multiple minimally rigid structures can be used to determine whether there exist rigid substructures that are consistent across random samplings of the data. If these re-appearing substructures exist, then there is stronger evidence that there exist relatively fixed regions or ‘structural invariants’ which can be more confidently in analyzed spatially.

Finally, we find that random graphs produced by applying the configuration model [27] to a chromosome conformation graph generally contain large rigid subgraphs as well. This suggests that the degree distribution of the graphs in these cases are linked to their rigidity.

## Conclusions

Recent chromosome conformation experiments provide an abundance of data which, even after applying several filtering strategies, still result in rigid embeddings for most of the budding yeast, fission yeast, and human genomes. This conclusion is independent of any particular algorithm for embedding a structure. The genome-wide graphs we studied are composed of one large rigid component using fewer than 2% of the edges. Additionally, we find that short-range interactions are crucial for maintaining the large rigid component.

As data for studying the three-dimensional structure of genomes under a variety of conditions becomes increasingly available, restricting spatial analysis to the high-confidence regions of these structures ensures that conclusions drawn from the structures are not artifacts of a lack of sufficient constraints. The algorithm proposed here efficiently identifies non-deformable, rigid substructures within chromosome conformation graphs by using a variety of results from rigidity theory that guarantee the construction of rigid graphs from rigid subgraphs. Graph rigidity is well-suited to assess the quality of chromosome conformation data since the experiments do not currently provide precise distances between chromosome locations, and graph rigidity does not depend on the precise values of the distances in a graph of distance constraints. Before performing computationally expensive embeddings of chromsosome conformation data, pre-processing data with the technique described in Algorithm 2 using any choice of filter quickly isolates regions of the genome for which a sufficient number of constraints exist for an embedding and these subgraphs serve as a basis for embedding chromosome conformation graphs in three dimensions.

## Methods

### Chromosome conformation experiments

Recent experimental methods for chromosome conformation [1-4] operate simultaneously on a million or more eukaryotic cells at the same stage of the cell cycle. The cells are chemically treated so that fragments of DNA bound to pairs of proteins near one another can be sequenced. This procedure results in a set of paired-end reads that can be mapped to pairs of chromosome locations that are near one another.

Depending on the experimental procedure, the pairwise interaction data is interpreted at different resolutions. Higher-resolution experiments consider the frequency of interaction between two DNA fragments directly while lower resolution experiments aggregate interactions between larger segments of DNA. Each pair of chromosome locations can be associated with a frequency of observed interaction, a statistical normalization of this frequency (e.g. divide frequencies by an expected genome-wide frequency), or an experimental normalization of this frequency. Table 2 lists the data sets that we use and the type of data they report.

**Table 2:** Chromosome conformation data sets

**Experiment**	**Genome**	**Resolution**	**Data provided**
Lieberman-Aiden et al.	Human	100,1000	R,C,SN
Duan et al.	Budding yeast	F,10	R,C,SN,EN
Tanizawa et al.	Fission yeast	20	R,SN,EN
Bau et al.	Human chr. 16	F	C

Reported information for various chromosome conformation data sets. Some data is analyzed at the restriction fragment length resolution (F) or at coarser resolutions (in kbp). Some experiments result in paired-end reads (R) that are deposited in an online database. Interaction frequency or counts (C), a statistical normalization of counts (SN), and experimental normalization of counts (EN) are also sometimes reported.

### Chromosome conformation graphs

A *chromosome conformation graph* encodes experimentally determined constraints between positions along one or more chromatin fibers. Formally, a conformation graph is a graph *G*=(*V*,*E*) where *V* is the set of centers of experimentally observed DNA fragments or larger segments of DNA, and the set of edges *E* corresponds to observed interactions and their frequency. Three of the four data sets we consider provide frequency data directly (Table 2). Tanizawa et al. instead provide experimentally normalized data, effectively dividing the observed counts by 20. Additional statistical normalization methods vary across publications, and there is no consensus yet for which normalization is appropriate to use.

An *augmented chromosome conformation graph* contains the vertices and edges of a chromosome conformation graph, but in addition contains vertices for chromosome fragments that were not observed to have any interaction partners and also includes edges connecting fragments that are adjacent to each other in the genome. Hence, the chromosome conformation graph contains only constraints measured by the experiments, while the augmented graph additionally contains a path representing each chromatin strand (Figure 3). The augmented graph explicitly incorporates the linear nature of the genome as packed chromatin [28]. Various methods to embed chromosome conformation data in three dimensions incorporate this type of constraint [2-4].

Table 3 summarizes the chromosome conformation graphs we created. Lieberman-Aiden et al. and Bau et al. perform experiments on lymphoblastoid cells (GM06690 and GM12878 respectively) as well as leukaemia cancer cells (K562). We use the 1Mb resolution for Lieberman-Aiden et al. since this is the resolution for which inter-chromosomal frequencies are provided. The chromosome conformation procedure described in Duan et al. involves two restriction enzymes: either HindIII or EcoRI paired with either MspI or MseI. To test the repeatability of their procedure, data is provided for both MspI and MseI. We use the frequency data from the experiment involving the HindIII and MspI restriction enzymes.

**Table 3:** Summary of chromosome conformation graphs

**Experiment**	**# Vertices**	**Max intra-chromosomal**	**Max inter-chromosomal**
		**frequency**	**frequency**
Lieberman-Aiden et al. GM06690	2,882	29,931	6,068
Lieberman-Aiden et al. K562	2,882	41,124	3,331
Duan et al.	4,193	4,683	107
Tanizawa et al.	619	35.25	13.75
Bau et al. GM12878	55	5,823	-
Bau et al. K562	55	13,686	-

Summary of chromosome conformation graphs used for testing embeddability in three dimensions. The frequencies in Tanizawa et al. are experimentally normalized, and Bau et al. focus on a 500kbp segment of human chromosome 16 as opposed to the entire genome.

### Preprocessing conformation graphs

Unfiltered chromosome conformation graphs are believed to contain noise due to (1) infrequent interactions and (2) short-range interactions. Although it is conceivable to consider all observed interactions simultaneously in a weighted fashion, existing embedding methods all directly filter input the data [2-4], and these methods preserve unusually high-frequency interactions given some genomic distance (e.g. [1-4]). Since there is no consensus yet on any particular filtering method, we systematically test whether a graph is rigid under a variety of filtering schemes: 

1. Frequency: remove *x*% of the lowest-frequency interactions. Existing filtering schemes keep the frequently occuring interactions while removing transient, potentially noisy ones.

2. Genomic distance: remove all interactions with endpoints separated by fewer than *x* kilobases. Existing filtering schemes also attempt to remove short-range interactions that may be a result of experimental noise.

3. Metric distance: remove all interactions that do not satisfy metric properties. Since existing embedding methods all employ a frequency-to-distance mapping [2-4], it is reasonable to remove constraints that violate metric properties of a graph. The set of interactions {*uv*} in the subgraph with lengths equal to the weighted shortest path between *u* and *v* satisfies the shortest path metric. To obtain this set, we calculate the shortest paths between the source and target nodes for all edges in the graph and keep only those edges whose length equals the shortest path length [29].

While we consider a variety of cases and data sets, to obtain an idea for the edge set sizes, the frequency cutoffs we consider for the genome-wide experiments are: 98.8, 99.0, 99.2, and 99.4%. For Duan et al. the edge set sizes for each respective cutoff are: 35892, 29910, 23928, 17946, and 11964. the edge set sizes for Lieberman-Aiden et al. are: 28426, 23921, 19328, 14689, and 10096 (healthy), 26798, 22681, 18471, 14053, and 9485 (cancer); the edge set sizes for Tanizawa et al. are: 2167, 1806, 1445, 1084, and 723.

Filtering methods 1 and 2 above allow us to systematically study the affect of removing low frequency interactions and short-range interactions so that we can identify which of these features contributes to the creation of rigid components (existing filtering methods combine the two properties making it difficult to isolate the cause of rigid components). Filtering method 3 is relevant since metrically consistent, low-error embeddings are desireable when embedding chromosome conformation data.

## Competing interests

The authors declare no competing interests.

## Authors contributions

GD and CK designed the algorithm, experimental setup, and wrote the paper. GD created the rigid components software and performed the experiments. Both authors read and approved the final manuscript.
